# Emotional response to illness in patients hospitalized with COVID-19 in russia

**DOI:** 10.1192/j.eurpsy.2021.289

**Published:** 2021-08-13

**Authors:** D. Dovbysh, M. Kiseleva

**Affiliations:** Pedagogy And Medical Psychology, Federal State Autonomous Educational Institution of Higher Education I.M. Sechenov First Moscow State Medical University of the Ministry of Health of the Russian Federation (Sechenov University), Moscow, Russian Federation

**Keywords:** coping with the disease, Depression, COVID-19, regulation of emotion

## Abstract

**Introduction:**

The situation of Covid-19 disease, associated with a high threat to life and uncertainty, had not only somatic, but also psychological consequences for most patients. Emotional reactions of patients to hospitalization and ways to cope with what is happening have become the subject of study in different countries.

**Objectives:**

To assess the severity of signs of depression and anxiety and to study the methods used to regulate the emotional state in patients with COVID-19 at the time of hospitalization.

**Methods:**

The study volunteered 127 hospitalized patients with Covid-19 (67 men (52.8%) and 60 women (47.2%), aged 19 to 77 years, who completed the following methods: Sociological questionnaire, Beck Depression Questionnaire, GAD-7, F-SOZU-22, CERQ, Dembo-Rubinstein self-assessment scales. The study was conducted from 04/25/2020 to 05/31/2020.

**Results:**

A quarter of patients showed pronounced signs of depression and anxiety (25.4% and 24.13%, respectively), with women having higher rates of depression (M = 8.76 and M = 6.32, p<0.01). Anova showed no significant differences in the response to the disease situation in patients of different age groups. Factor analysis made it possible to identify 3 patterns of emotional coping with the disease: «positive decision oriented», «fixed on negative experiences», «accusers» (The resulting factor solution explains 69% of the dispersion). None of the identified patterns were associated with a significant reduction in signs of depression and anxiety.

**Conclusions:**

Despite the duration of the pandemic, there is still no specific pattern of effective coping with these experiences for patients.

**Disclosure:**

No significant relationships.

